# Supplementary material for: Initiation of HIV neutralizing B cell lineages with sequential envelope immunizations
Source: Nat Commun. 2017 Nov 23;8:1732. doi: 10.1038/s41467-017-01336-3 (PMC5701043; doi:10.1038/s41467-017-01336-3)
Supplement: Supplementary file 1 — Supplementary Information [file 41467_2017_1336_MOESM1_ESM.pdf]

SUPPLEMENTARY FIGURES

A. CH505 Env-reactive memory B cells (%)

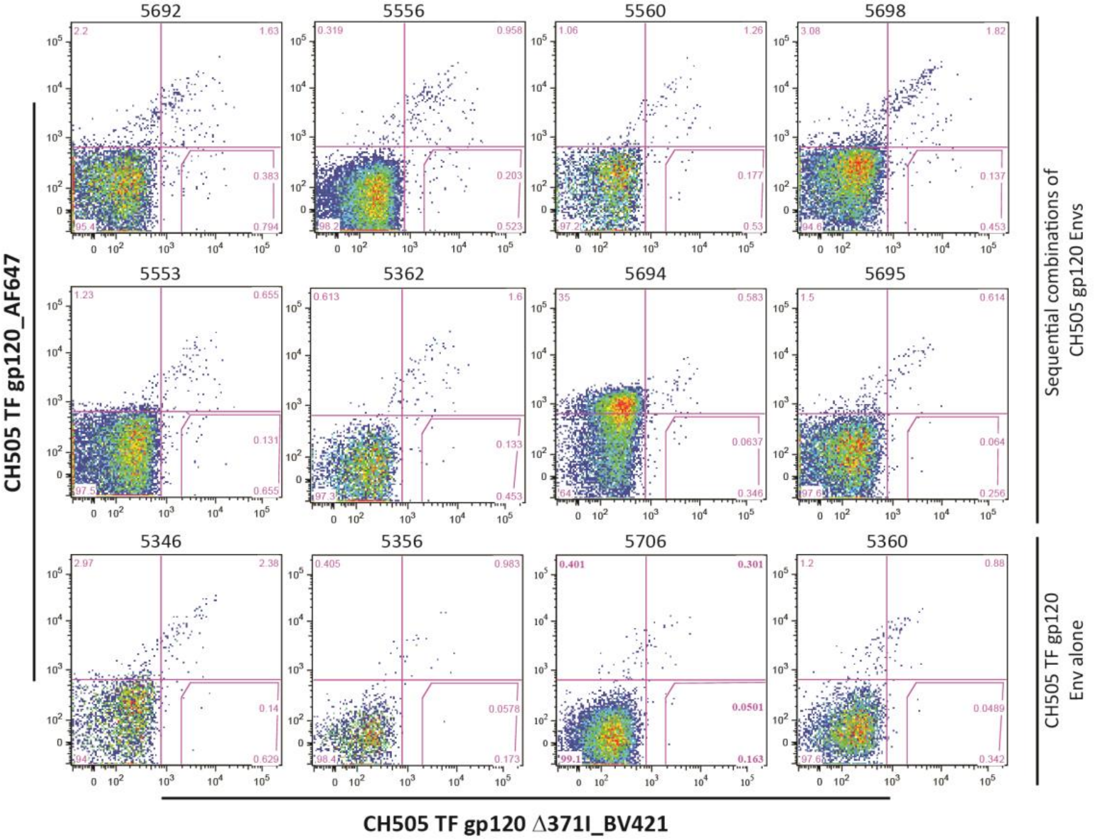

B. CH505 differential binding memory B cells

Animal ID	Memory B cells (count)	CH505 TF gp120 + CH505 TF gp120 Δ371I- (count/ percent)
Sequential combinations of CH505 gp120 Envs		
5692	7302	28 (0.38%)
5556	13,772	28 (0.2%)
5560	3966	7 (0.18%)
5698	9487	13 (0.14%)
5553	11,458	15 (0.13%)
5362	3749	5 (0.13%)
5694	10,985	7 (0.06%)
5695	7818	5 (0.06%)
CH505 TF gp120 Env alone		
5346	2862	4 (0.14%)
5356	1730	1 (0.06%)
5706	7979	4 (0.05%)
5360	4091	2 (0.05%)

C. Gating hierarchy

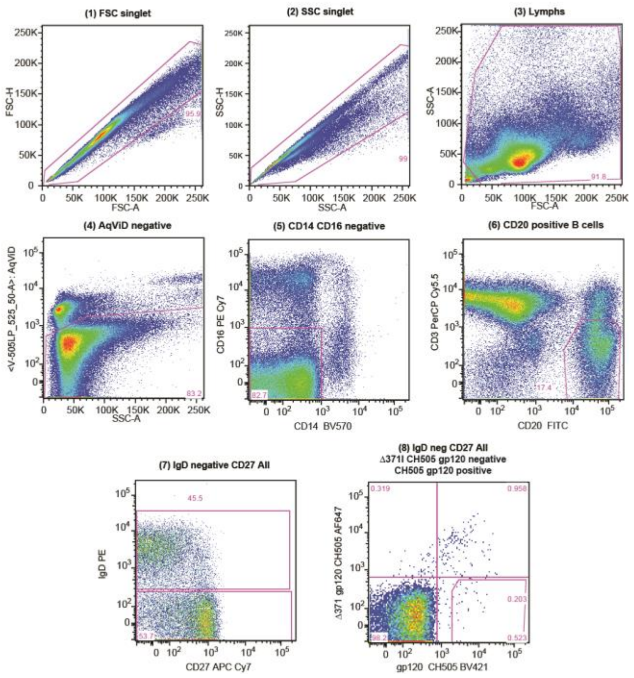

Supplementary Figure 1 continues on next page

Supplementary Figure 1. Representative flow cytometric plots with the gating strategy to enumerate CH505 gp120 Env-reactive memory B cells. **(A)** IgD-negative, CD27-All memory B cells from blood were analyzed for CH505 differential-binding memory B cells that reacted with CH505 TF gp120, but not CH505 TF gp120 Δ371I mutant protien; gated cells in lower right quadrant of the flow plots. **(B)** Quantification of total memory and CH505 differential-binding memory B cells for each plot shown in panel A. (A-B) These data represent the highest frequency of CH505 differential-binding memory B cells above background at 2 weeks post 3rd immunization (week 14) for animals immunized with CH505 TF gp120 Env monomer alone and sequential CH505 gp120 Env monomers. **(C)** Flow cytometry gating hierarchy of events (1-8) captured to cells analyzed. Shown are representative plots from one macaque, 5556.

A. mAbs (CH505 differential-binders)

Vaccination regimens	Antibody ID	Immunogenetics				
		Heavy chain				
		V_H	D_H	J_H	Mut. Freq. (%)	CDR3 length (aa)
CH505 sequential Envs	DH731**	1-E	6-19	4-1	7.6	18
	DH732**	1-G	1-8	2-1	6.6	13
	DH733	3-A	2-16	6-1	1.4	16
	DH734**	3-D	3-5	4-1	3.8	17
	DH735**	3-K	2C-7	4-1	6.9	15
	DH566	3-Q	6-19	4-1	5.6	18
	DH736**	3-Q	2C-7	5-1	3.8	14
	DH737**	3-V	2-13	4-1	7.5	16
	DH738	4-B	2C-16	2-1	2.4	14
	DH739	4-D	3-12	4-1	4.1	17
	DH740*	4-D	7-23	5-1	6.9	16
	DH741**	4-F	3-21	6-1	3.5	20
	DH742*	4-G	2C-13	5-1	4.5	16
	DH743*	4-G	2-1	5-1	2.1	16
	DH744**	4-H	3C-12	5-1	7.9	18
	DH522.1**	4-J	3-12	4-1	3.4	17
	DH522.2	4-J	3-12	4-1	4.5	17
	DH745**	4-L	3-12	5-1	1.0	19
	DH746	4-L	3-12	1-1	3.1	18
CH505 TF alone	DH747	1-E	2-16	5-1	1.4	21
	DH359**	3-A	2-1	4-1	2.1	17
	DH748*	3-A	3-5	4-1	2.4	16
	DH749**	3-J	3-5	5-1	9.0	19
	DH750	3-O	3-14	4-1	2.8	14
	DH751.1**	3-V	1-8	4-1	4.1	16
	DH751.2*	3-V	1-8	4-1	5.1	16
	DH360	3-V	3-12	4-1	3.1	16
	DH362	3-V	3-12	4-1	2.7	16
	DH752*	4-M	3-12	5-1	4.5	21

B. CH505 differential-binding profile

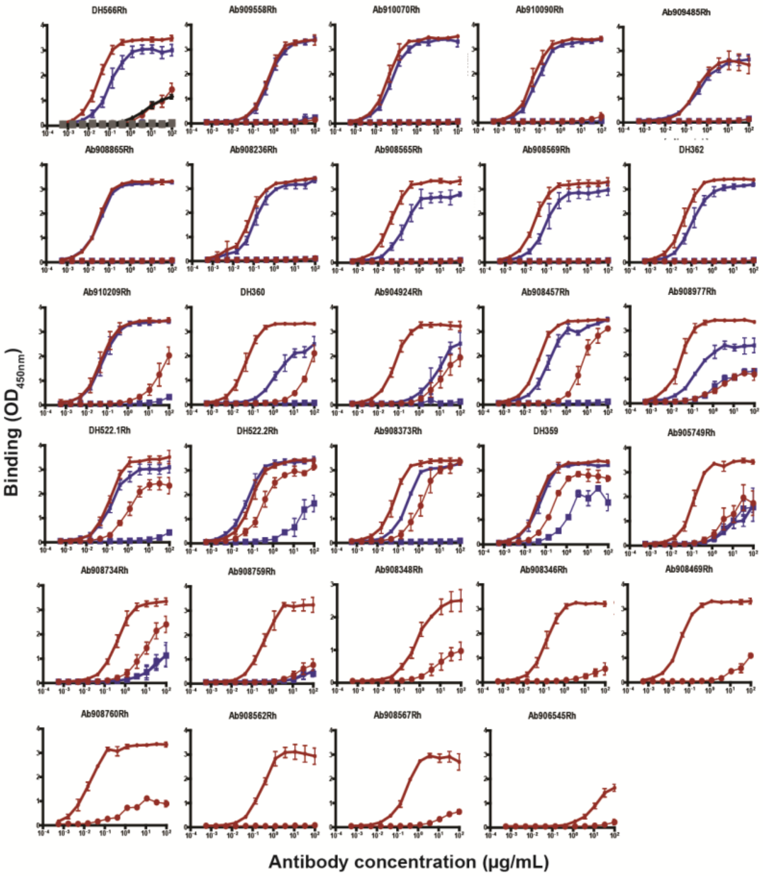

C. Autologous Env gp120 binding

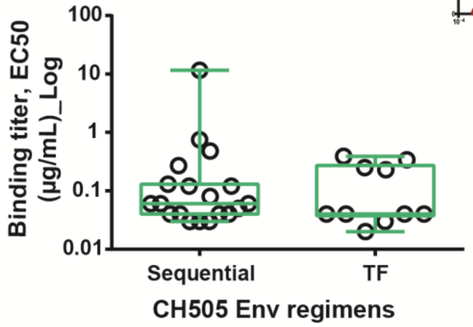

D. Autologous neutralization (CH505.w4.3)

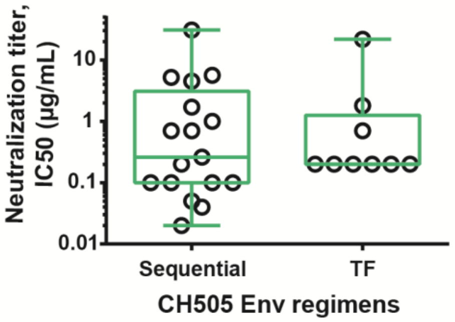

E. Heterologous neutralization

Antibodies	CH103	F105	DH522.1
# Viruses	196	206	199
**Total Viruses Neutralized**			
IC50 <50ug/ml	104	14	14
IC50 <10ug/ml	69	12	10
IC50 <1.0ug/ml	19	5	2
IC50 <0.1ug/ml	3	1	0
IC50 <0.01ug/ml	0	0	0
**% Viruses Neutralized**			
IC50 <50ug/ml	53	7	7
IC50 <10ug/ml	35	6	5
IC50 <1.0ug/ml	10	2	1
IC50 <0.1ug/ml	2	0	0
IC50 <0.01ug/ml	0	0	0
Median IC50	6.84	2.61	4.61
Geometric Mean	4.54	1.65	3.87

F. Chimeric antibodies profile

Antibodies	CH505 TF gp120 binding, EC50 (µg/ml)	CH505.w4.3 neutralization, IC50 (µg/ml)
CH103VH + DH522VL	35.3	>50
DH522VH + CH103VL	nb	>50
DH522VH + F105VK	nb	>50
F105VH + CH103VL	nb	>50
F105VH + DH522VL	18.1	>50
CH103	0.06	**0.3**
DH522	0.08	**0.3**
F105	0.11	>50

nb. no binding

Supplementary Figure 2 continues on next page.

Supplementary Figure 2. Characteristics of representative CH505 differential-binder antibodies isolated from CH505 envelope (Env)-vaccinated macaques. **(A)** Immunogenetics of 29 representative monoclonal antibodies (mAbs). Of the 29 mAbs selected, 19 were initially screened for neutralization of CH505.w4.3 as purified IgG based on sample availability and 13/19 were CH505.w4.3 neutralization-sensitive; *screened as purified IgG for CH505.w4.3 neutralization and **positive neutralization detected. An additional 16/29 antibodies were selected based on representative immunogenetics. Antibody immunogenetics were inferred using the Cloanalyst software program. **(B)** CH505 differential-binding profile to autologous (CH505 TF gp120 – red line, closed circles; CH505 TF gp120 Δ371I – red line, open circles) and heterologous (YU2 gp120 – blue line, closed squares; YU2 gp120 D368R – blue line, open squares; RSC3 gp120 – black line, closed diamonds; RSC3 gp120 Δ371I – black line, open diamonds) Envs in replicate ELISAs (2-4x). The data shown represent the average binding across multiple assays; error bars represent standard deviation for ≥3 assays and standard error of the mean for 2 assays. **(C)** Binding titer of CH505 differential binder mAbs to CH505 gp120 Envs. Binding was determined by ELISA and reported as EC50 (µg/ml) from a representative assay. **(D)** Neutralization titer of CH505 differential binder mAbs against CH505.w4.3 virus performed in TZM-bl cells and reported as IC50 (µg/ml) from a representative assay. **(C-D)** The titers are shown via box and whisker plots; box (median, and lower and upper quartile ranges) and whiskers (minimum and maximum). **(E)** Heterologous virus neutralization profile of DH522 performed in TZM-bl cells against a panel of 199 heterologous viruses at an independent research site (Vaccine Research Center, NIH). CD4 binding-site antibodies, CH103 and F105, were tested for comparison. A summary of the percent viruses neutralized and mean percent neutralization titers for all 3 mAbs are shown. See supplementary methods for list of viruses neutralized by DH522. **(F)** Binding specificity and neutralization profile of chimeric CH103, F105 and DH522.1 mAbs. Binding titers from a representative ELISA are reported; nb – no binding. CH505 TF gp120 Envs were screened for VH/VL chimeric-antibody binding. Titers for neutralization performed in TZM-bl cells against autologous tier 1 CH505.w4.3 virus from a single assay are reported. Both DH522.1 and CH103 mAbs neutralized C.6644, D.57128, AG.DJ263, C.MW965, B.SF162 and M.CON-S isolates, F105 neutralized D.57128 and B.SF162 isolates, and F105VH+DH522VL chimeric mAb weakly neutralized B.SF162 (IC50, 9 µg/mL); no neutralization breadth was observed for chimeric mAbs.

A. Env gp120 binding

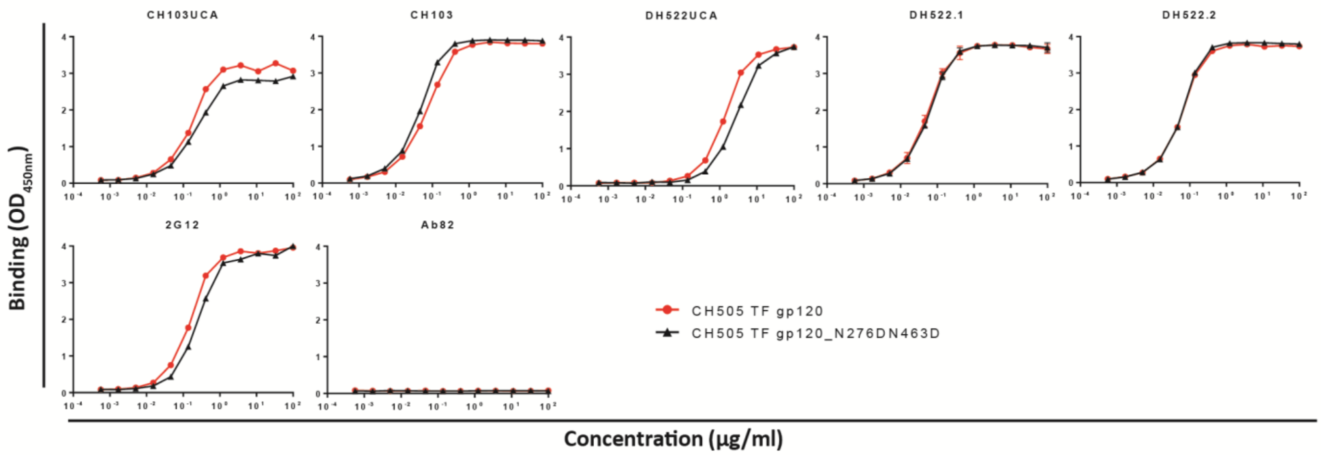

B. HIV-1 neutralization

Antibody ID	IC50 (µg/ml) in TZM-bl cells				
	MuLV	CH505 TF	CH505TF.gly4	426c	426c.N276D.N460D.N463D
CH103UCA	>50	>50	>50	>50	>50
CH103	>50	3.4	<0.023	>50	4.7
DH522UCA	>50	>50	>50	>50	>50
DH522.1	>50	>50	>50	>50	>50
DH522.2	>50	>50	>50	>50	>50

C. Glycan binding

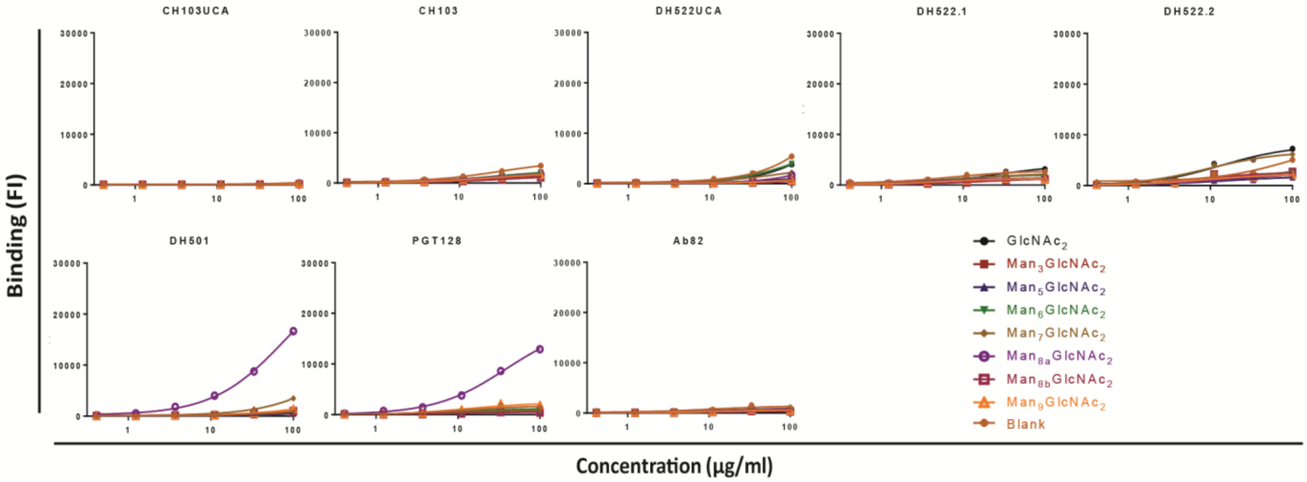

Supplementary Figure 3. Impact of HIV-1 Env glycosylation and access to the CD4 binding-site epitope by human and macaque antibodies. **(A)** ELISA binding of human and macaque antibodies to recombinant gp120 CH505 Envs; wildtype and mutant Env with glycans deleted at position 276 and 463 surrounding the CD4 binding-site (1). Binding titers are reported as EC50 (µg/ml) and are representative of 3 assays. HIV-1 Env and glycan-reactive Ab 2G12 was used as a positive control. **(B)** Neutralization titers (IC50 in µg/mL) for HIV-1 pseudoviruses containing wildtype CH505 or 426c Env strain, and mutant Envs with a deletion of glycans in the vicinity of the CD4 binding-site (197, 276, 362, 462) as previously described (1). Neutralization was performed in TZM-bl cells in a single experiment. Neutralization positivity cutoff was 50 µg/ml; positive neutralization titers are shown in red font. **(C)** Binding to glycans by CH103 and DH522 lineage antibodies was measured in a luminex microsphere assay (2). DH501 and PGT128, V3 glycan neutralizing antibodies, provided references for positive binders. Anti-influenza antibody, Ab82, was used as a negative control.

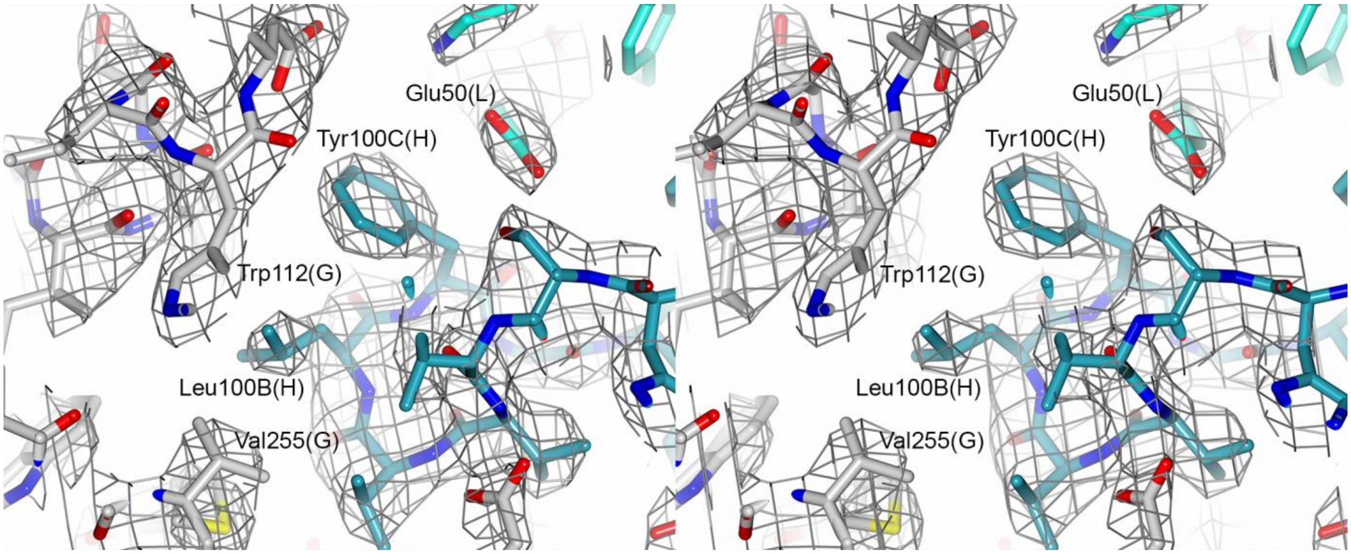

Supplementary Figure 4. Stereo image of the electron density map for the DH565-gp120 core complex crystal structure. The heavy and light chains of DH522.2 Fab are depicted with dark and light cyan carbon atoms, respectively, and deglycosylated chimeric B.YU2 gp120 core with light grey carbon atoms as in Fig S4B. The 2Fo-Fc electron density map is shown as a grey mesh at a 1.0 sigma level contour. This figure was generated with CCP4 Molecular Graphics (3).

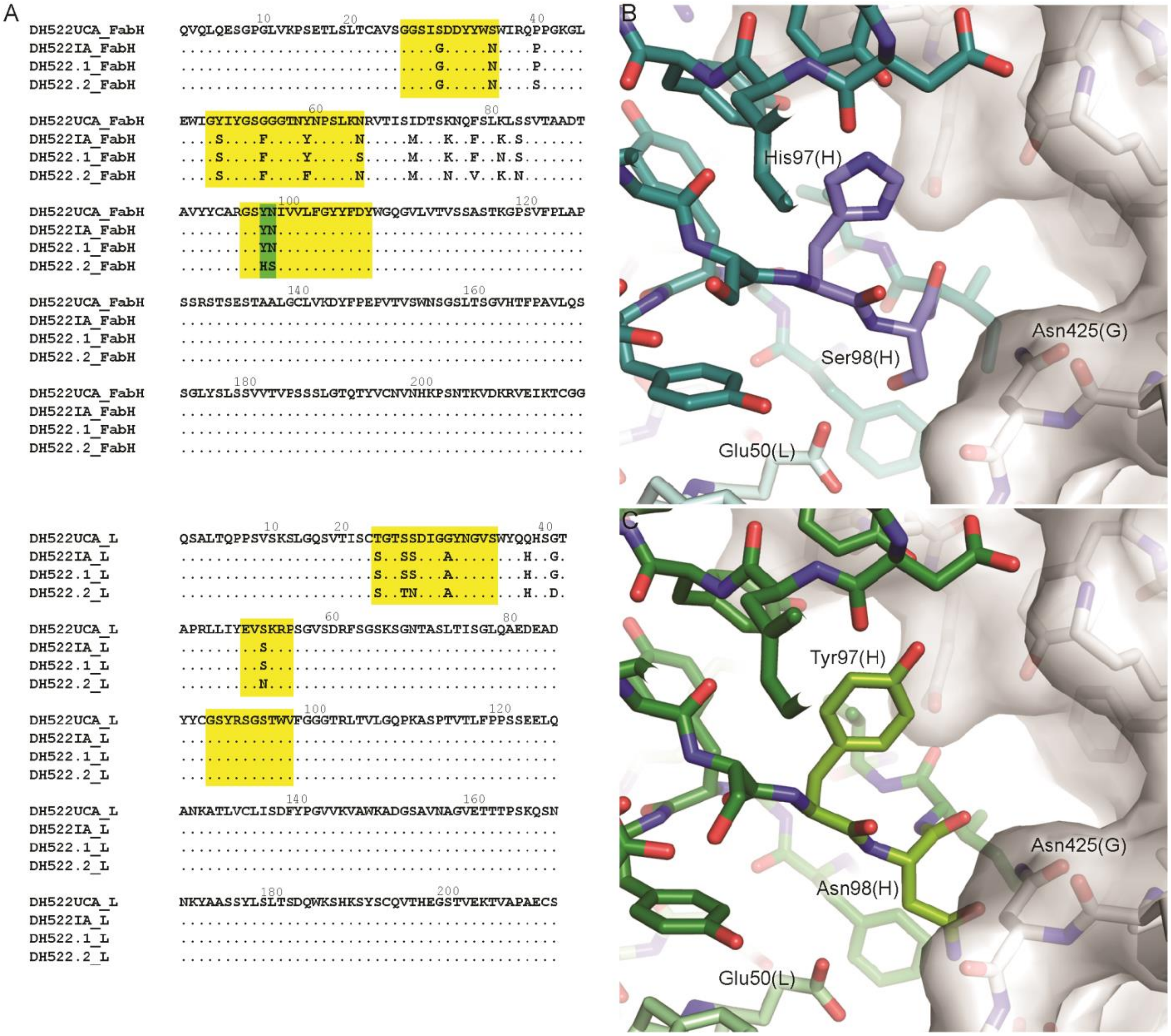

Supplementary Figure 5. Structural comparison within the DH522 lineage. **(A)** Multiple sequence alignments of the heavy (above) and light (below) chains of antibodies in the DH522 lineage are shown with CDRs boxed in yellow and a noted distinguishing feature of DH522.2 boxed in green. **(B)** The DH522.2 complex structure (above) showed the HCDR3 His-Ser motif (residues 97-98) with an optimal steric fit in the antibody-antigen interface near gp120 residue Asn425 compared to a Tyr-Asn motif for the superimposed DH522.1 model (below). Nearby residue Glu50 on the light chain is also labeled as it may contribute to steric fit in the vicinity

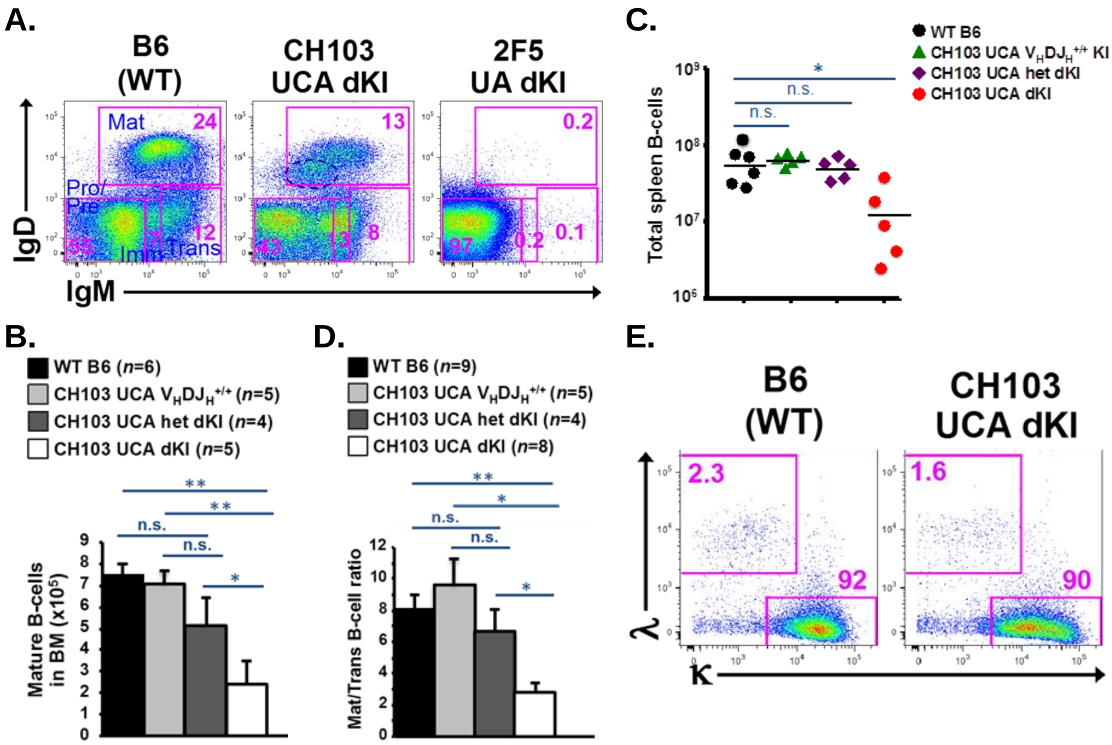

Supplementary Figure 6. B-cell development in various naïve CH103UCA knock-in strains. **(A-B)** Flow cytometric analysis of central B-cell development. **(A)** Distinct stages of negative B-cell selection in CH103 and 2F5 knock-in mice. Shown are cytograms of bone marrow B-cell development naïve CH103UCA double knock-in ($V_HDJ_H^{+/+}$ x $V_lJ_l^{+/-}$) mice, which are subjected to partial deletion and anergy at the 2nd tolerance checkpoint, compared to normal development in wild type C57BL/6 gender-matched littermates (WT B6) or in relation to the more profound and early (1st tolerance checkpoint) deletion seen in 2F5 UA double knock-in mice, which exhibit a marked developmental blockade at the pre-B->immature B-cell transition. Numbers indicate percentages in Progenitor/precursor (Pro/pre), Immature (Imm), Transitional (Trans), and recirculating mature (Mat) subsets. Data are gated on live, total B (CD19+B220+) cells. Note the subset of mature B-cells (demarcated by the blue, dotted oval) with lowered BCR densities; this subset corresponds to the Env+ (non-edited) mature B-cell subpopulations indicated in Figure 6A. **(B)** Graphical summary of absolute numbers of recirculating mature B-cells in bone marrow of naïve CH103UCA $V_HDJ_H^{+/+}$ knock-in (“HC only”), het double knock-in ($V_HDJ_H^{+/-}$ x $V_lJ_l^{+/-}$), double knock-in strains, relative to WT B6 controls. Numbers were calculated based on flow cytometric fractionation of Mat B-cells, defined as live singlet, $B220^+CD19^+IgD^{hi}IgM^{lo}$ bone marrow lymphocytes. **(C-E)** Flow cytometric analysis of peripheral B-cell development in CH103UCA HC only, het double knock-in, or double knock-in mice, relative to WT B6 controls. **(C)** Graphical summary of total numbers of peripheral B-cells (defined as live, singlet, lymphocyte, B220+CD19+ splenocytes). Individual mice are denoted by distinct colored shapes for each strain, and means are represented by black bars. **(D)** Graphical summary of Mature follicular (Mat) / Transitional (Trans) peripheral B-cell ratios. Mat B-cells=live singlet, lymphocyte-gated, B220+CD19+CD93-CD21+CD23+ splenocytes; Trans B-cells=live singlet, lymphocyte-gated, B220+ CD19+ CD93+ CD21-CD23- splenocytes. **(E)** Surface kappa and lambda LC expression in CH103UCA double knock-in mice. Shown are cytograms (representative of two experiments) gated on total splenic B-cells (live B220+CD19+), with numbers in magenta indicating percentages positive for surface LC k or l_{1-3} LC expression. *$p \leq 0.05$; **$p \leq 0.005$; n.s.=not significant, two-tailed Student’s *t*-test. Abbreviations: UCA, unmutated common ancestor; HC, heavy chain; LC, light chain; het, heterozygous.

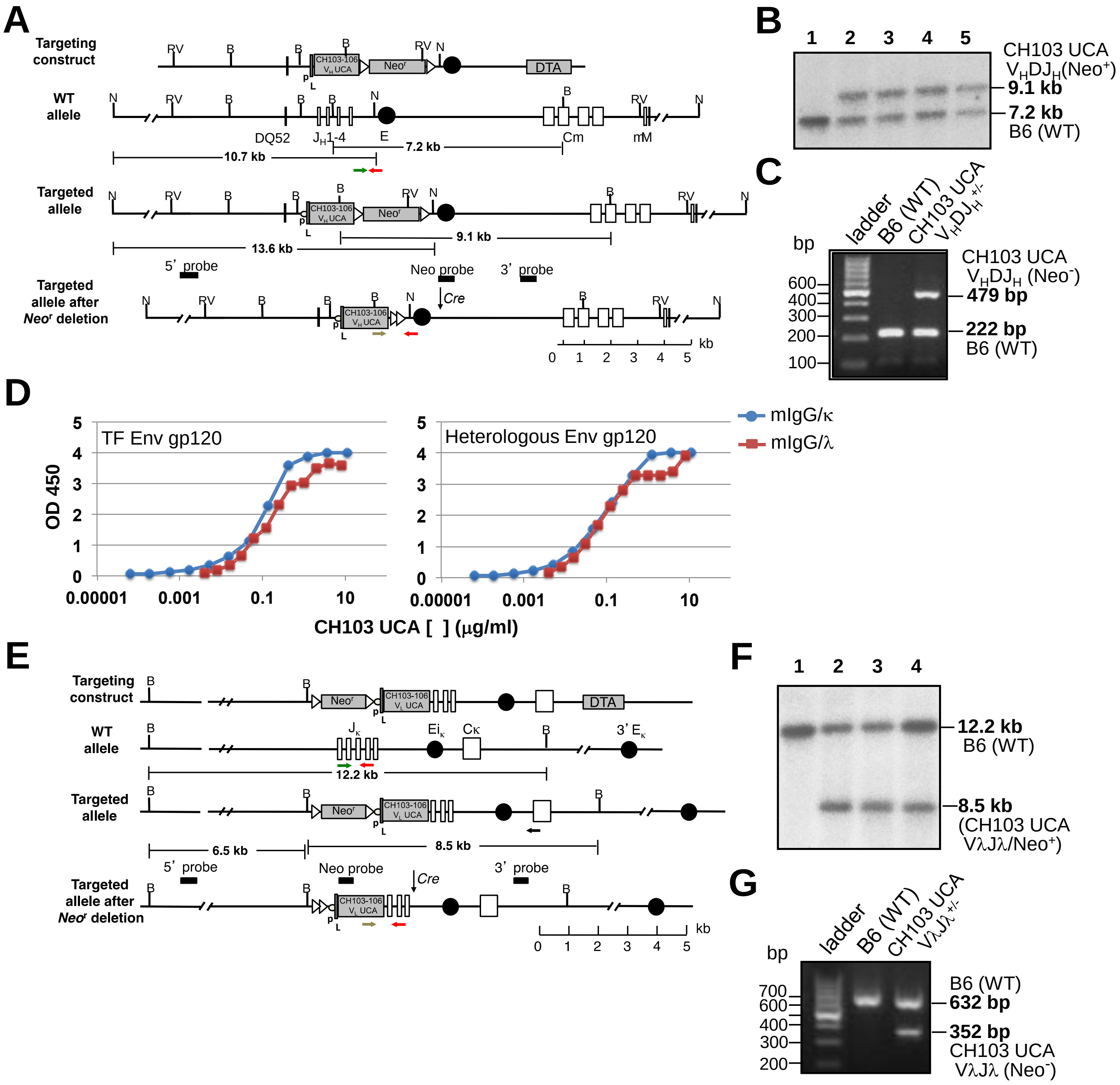

Supplementary Figure 7. *In vivo* targeted replacement of the mouse IgH and Igk loci with the human CH103 UCA V_HDJ_H and CH103 UCA $V\lambda J\lambda$ rearrangements, respectively. **(A)** Site-directed strategy used to knock in the CH103 UCA V_HDJ_H rearrangement, based on previous methods (3). The V_HDJ_H UCA expression cassette comprises a J558 H10 family promoter (p), an H10 split leader (L), and the CH103 UCA V_HDJ_H rearrangement. The IgH intronic enhancer (E) is represented by a circle and loxP sites are depicted as triangles, probes used to verify homologous recombination events are shown as black bars, and genotyping primers are denoted by red, green and gray arrows. B, *Bam*HI; RV, *Eco*RV; N, *Nsi*I. **(B)** Confirmation of targeted insertion of the CH103 UCA V_HDJ_H rearrangement into the mouse Igh locus. Representative Southern blot of genomic DNA from parental (lane1) and four recombinant neo+ ES cell clones harboring the targeted CH103 UCA V_HDJ_H (lanes 2-5). **(C)** Representative PCR of tail DNA from a CH103 UCA $V_HDJ_H^{+/-}$ KI mouse after *in vivo cre*-mediated deletion of the Neo^r cassette. PCR primers specific for WT or targeted alleles=green and gray arrows, respectively; primer

common to both alleles=red arrow. **(D)** Functional analysis of chimeric, recombinant CH103 UCA antibodies. mIgG1/κ and mIgG1/λ (made with Cκ and Cλ regions, respectively) show comparable Env gp120 binding as assessed by ELISA (4). CH505 lineage transmitted/founder virus (TF Env gp120) or a heterologous virus, 635211 D11 (heterologous gp120). mIgG1/κ and mIgG1/λ were made as previously described (3). Briefly, the CH103 UCA V_HDJ_H rearrangement was ligated to mouse Cγ1, and the CH103 UCA VλJλ rearrangement was fused to either mouse Cκ or Cλ and all three were cloned into pCDNA 3.1. After co-expression in 293T cells, resulting recombinant antibodies (mIgG1/κ and mIgG1/λ, respectively) were purified by standard methods. (**E)** Site-directed strategy used to knock in the CH103 UCA VλJλ rearrangement, based on previous methodologies (5, 6). The UCA VλJλ expression cassette comprises a VκOx1 promoter (p), a VκOx1 split leader (L), and the CH103 UCA VλJλ rearrangement. Annotation similar to (A). **(F)** Representative Southern blot of genomic DNA from parental (lane1) and recombinant neo+ ES cell clones harboring the targeted allele (lanes 2-4). **(G)** Representative PCR showing of tail DNA from a CH103UCA $V\lambda J\lambda^{+/-}$ KI mouse after *in vivo cre*-mediated deletion of the Neo^r cassette. PCR primers for WT or targeted alleles, green and gray arrows, respectively; primer common to both alleles, red arrow.

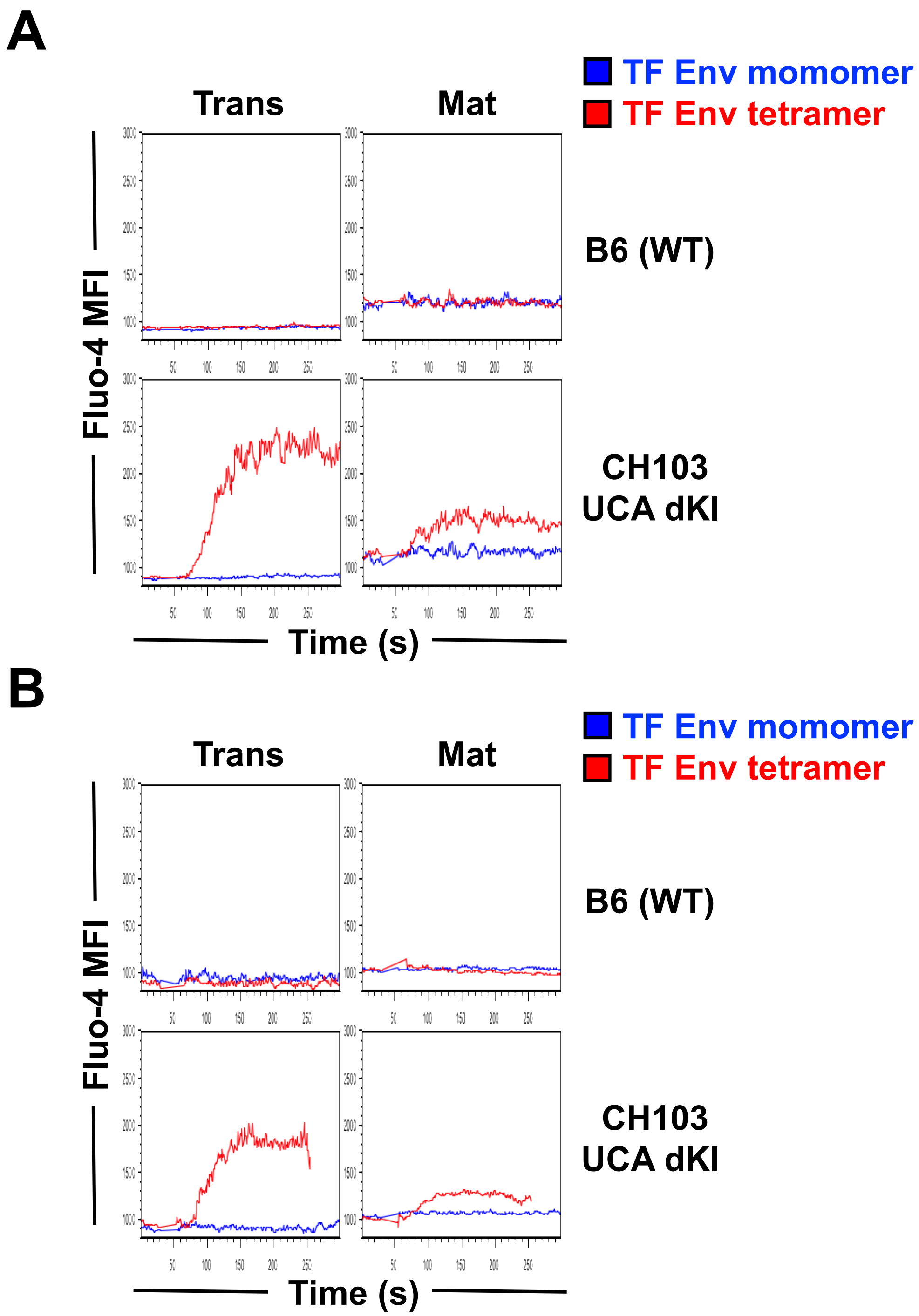

Supplementary Figure 8. *Ex vivo* functional analysis of signaling responses to TF Env by CH103UCA double knock-in (dKI) transitional and mature B-cells. Shown are calcium flux responses of CH103UCA double knock-in or C57BL/6 (B6 WT) littermates. Prestained bone marrow transitional ($IgM^{hi}IgD^{lo}$) or recirculating mature ($IgM^{lo}IgD^{hi}$) B-cells **(A)** or splenic transitional ($CD93^{+}CD21^{-}CD23^{-}$) or follicular mature ($CD93^{-}CD21^{+}CD23^{+}$) B-cells **(B)** were loaded with Fluo-4, and either baseline levels of Ca^{++} release prior to stimulation, or those in response to BCR-saturating levels (100 nM) of monomeric or tetrameric forms of the CH103UCA-directed TF Env priming immunogen (denoted by green arrows) were measured as Fluo-4 mean fluorescence intensities on the y-axis, for the indicated times (on the x-axis). Data is representative of two independent experiments. Abbreviations: UCA, unmutated common ancestor; dKI, double knockin; TF, transmitted-founder.

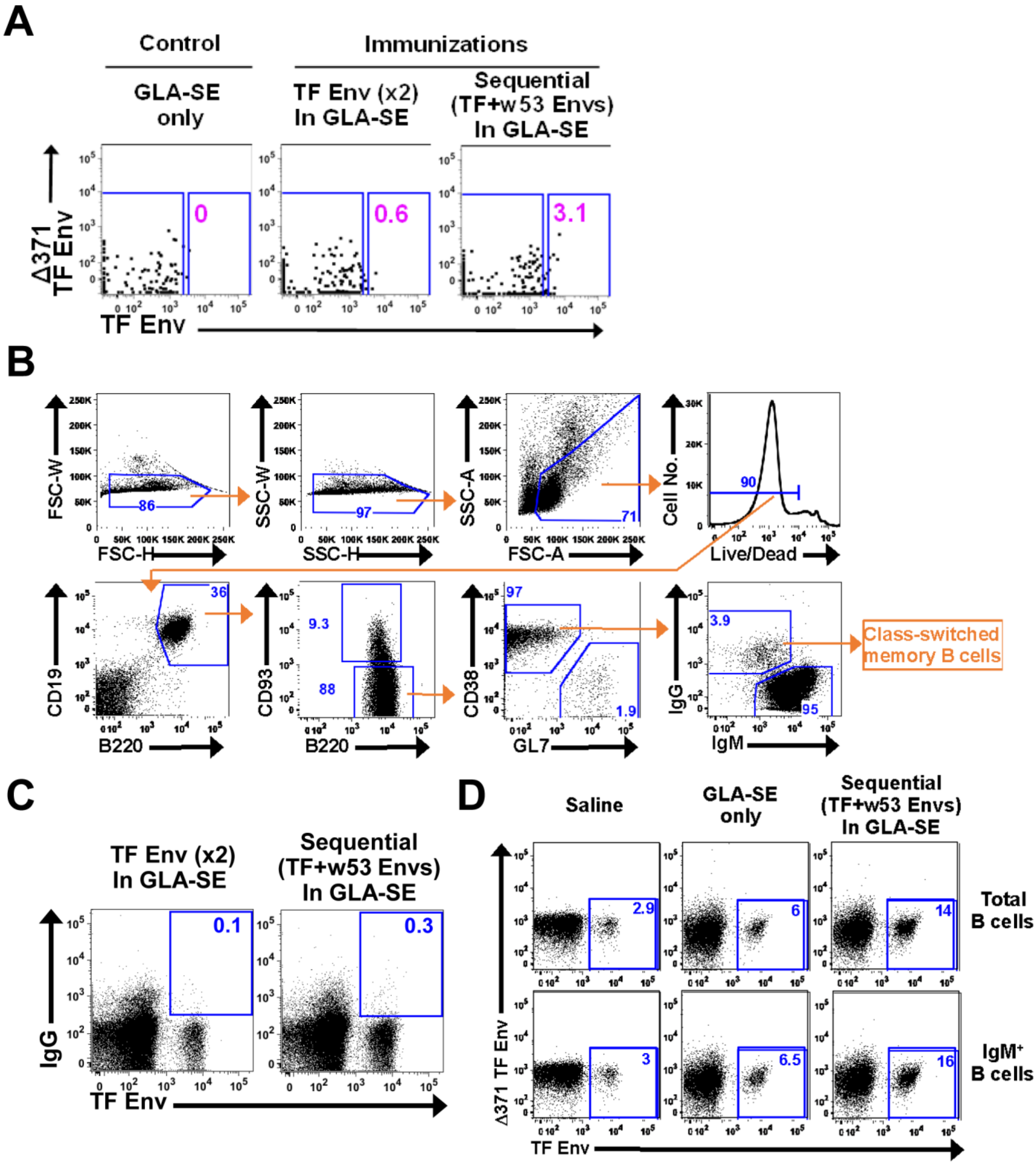

Supplementary Figure 9. Characterization of B-cell responses to TF+week 53 envelope sequential immunization in CH103UCA het double knock-in mice. **(A)** Class-switched memory B-cell responses in draining lymph nodes (dLNs) of CH103 het dKIT/F+week 53 mice activated by envelope (Env) immunization. Shown are flow dot plot histograms of IgG+ memory B-cells taken from dLNs 10d after second immunizations, from groups administered with GLA-SE only or Env-immunized (twice with TF Env or sequentially with TF+week 53 Env). Numbers denote frequencies of TF Env differential-binding (lineage-specific) memory clones, i.e. fractionated based on a mutant (Δ371) TF Env- and wild type (WT) TF Env+ gating strategy, within the total IgG+ memory B-cell fraction (the latter defined as shown in (B). **(B)** Representative flow cytometry gating strategy used to further analyze class-

switched memory B-cells phenotyped and/or sorted in Figures 7A, C-F and phenotyped in Supplementary Figure 9A, all based on their Env reactivities. Doublets were excluded using forward scatter gating, from which a lymphocyte cell gate was drawn, followed by selection for total live cells by Live/Dead Infrared- gating, ensued by selection for total B-cells via CD19+ and B220+ gating, memory B-cell selection by CD93- and CD38+ gating, and finally gating on the IgG+ fraction for class-switched clones. Example shown is from a sequentially-immunized CH103 het double knock-in mouse. **(C)** TF Env-reactive, IgG-switched memory B-cells (indicated by a blue gate) induced in immunized CH103 het double knock-in mice, within the total memory (B220+CD19+CD93-CD38+) B-cell fraction. Numbers indicate their overall frequencies amongst all memory B-cells. **(D)** Expansion of IgM+ splenic B-cell compartment in Env and/or adjuvant-administered CH103UCA het dKI mice. FACS histograms of TF Env differential reactivity in total (singlet, live CD19+B220+) splenic B-cells (top row) or IgM-gated total splenic B-cells (bottom row) from control (saline-injected or GLA-SE adjuvant-only injected) and immunized (TF+week 53 Env in GLA-SE; sequentially-immunized) CH103 UCA het double knock-in mice, harvested 10d after 2nd immunizations. Numbers indicate frequencies of TF Env differential-binding total B-cells.

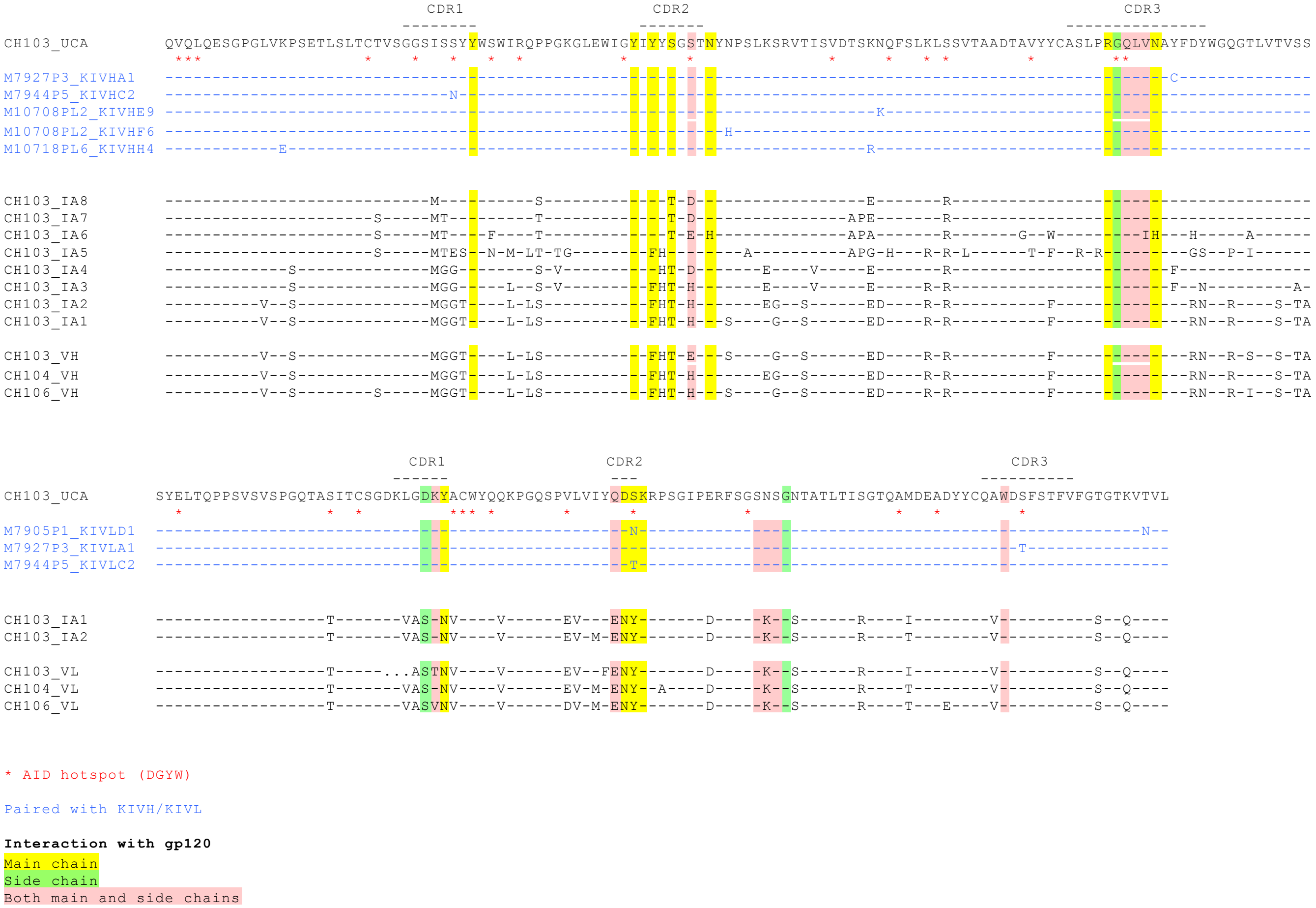

Supplementary Figure 10. Amino acid alignments of CH103 lineage-specific single IgG^{+} memory b- cell clones recovered from immunized CH103UCA het double knock-in mice. Mutations in double knock-in positive clone sequence relative to the CH103 UCA are indicated in blue lettering, while those in CH103 lineage intermediates and matured broadly neutralizing antibodies isolated from the HIV-1 chronically-infected subject CH505 (4), are shown in black lettering, with shared residues that interact with Env indicated by yellow, green, and red highlights, indicating gp120 main chain, side chain, and main+side chain contacts, respectively. ⁎ represents AID hotspots, DGYW (5). Abbreviations: het, heterozygous; UCA, unmutated common ancestor; TF, transmitted-founder; wk, week

SUPPLEMENTARY TABLES

Supplementary Table 1. Data collection and refinement statistics.

	DH522.1	DH522UCA	DH522IA	DH522.2	DH522.2-gp120 core
**Data collection***					
Space group	P 31 2 1	P 31 2 1	P 31 2 1	C 2 2 21	P 21 21 21
Cell dimensions					
a, *b*, *c* (Å)	70.68, 70.68, 169.84	68.52, 68.52, 178.38	68.71, 68.71, 176.18	70.07, 79.22, 184.75	50.85, 71.97, 252.23
α, β, γ (°)	90, 90, 120	90, 90, 120	90, 90, 120	90, 90, 90	90, 90, 90
No. of Reflections	191456	517740	84755	120959	122672
No. of Unique Reflections	33888 (1670)	49650 (2456)	20786 (1035)	30338 (1531)	25631 (1275)
Resolution (Å)	50.0-2.00 (2.03-2.00)	50.0-1.75 (1.78-1.75)	50.0-2.30 (2.34-2.30)	50.0-2.10 (2.14-2.10)	50-2.70 (2.75-2.70)
R_{sym} or R_{merge}	0.07 (0.44)	0.06 (0.34)	0.12 (0.56)	0.11 (0.39)	0.14 (0.80)
$\langle I/\sigma I \rangle$	18.4 (3.0)	36.7 (6.8)	13.8 (2.8)	12.1 (2.4)	13.5 (2.2)
Completeness (%)	99.7 (99.6)	99.2 (100)	93.1 (95.4)	96.3 (97.3)	98.6 (100)
Redundancy	5.6 (5.2)	10.4 (8.3)	4.1 (4.3)	4.0 (4.1)	4.8 (4.4)
**Refinement**					
Resolution (Å)	41.6-2.0 (2.05-2.00)	42.0-1.75 (1.79-1.75)	23.2-2.29 (2.35-2.29)	24.3-2.10 (2.16-2.10)	49.85-2.71 (2.78-2.71)
No. reflections	32746 (2017)	49175 (3230)	20038 (1096)	27150 (1772)	25574 (1576)
R_{work}/R_{free} (%)	19.2/23.6 (21.3/28.2)	16.7/20.2 (20.7/22.9)	18.1/26.7 (23.0/32.5)	17.4/23.0 (21.5/27.7)	20.1/26.5 (25.8/33.9)
No. atoms / Average *B* values (Å^2)					
Protein	3216 / 26.60	3291 / 37.23	3267 / 50.46	3239 / 40.49	4853 / 55.91
Solvent	161 / 25.08	412 / 45.61	147 / 49.83	263 / 44.09	17 / 36.36
Glycan	n/a	n/a	n/a	n/a	84 / 93.41
R.m.s deviations					
Bond lengths (Å)	0.007	0.007	0.008	0.008	0.008
Bond angles (º)	1.182	1.102	1.133	1.174	1.267
**Model Validation**					
Ramachandran favored (%)	96.7	97.2	97.0	97.4	94.1
Ramachandran outliers (%)	0.2	0.0	0.0	0.0	0.3

Each dataset was reduced from images collected on a single crystal.
*Highest resolution shell is shown in parentheses.

Supplementary Table 2. Neutralization activity of serum antibodies from immunized CH103UCA het double knock-in mice**.** Shown are the reciprocals of geometric mean titers (GMTs) required for ID50 neutralization, as tested via the TZM-bl neutralization assay. Positive neutralization (defined as >2 the minimal cutoff value), is highlighted in yellow. Naïve C57BL/6 WT animals (representing age, gender, strain background & cage-matched mice) were used as controls for non-specific background neutralization activity found in mouse serum. Abbreviations: UCA, unmutated common ancestor; het, heterozygous.

Strain / Vaccine Regimen	Time Point	HIV -1 isolates (serum antibody neutralization, ID50 GMTs)						
		Tier 2, Clade C	Tier 1b, Clade C	Tier 1, Clade C	Tier 1b, Clade C	Tier 1, Clade B	Tier 1b, Clade B	Tier 2, Clade D
		CH505 TF	CH505. w4.3	MW965	C.6644	SF162	SS1196	D.57128
CH103 UCA het double knock-in/ GLA-SE only control (*n*=4)	Pre-bleed	<80	111	89	<80	<80	93	96
	Post-immune 1 & 2 (pooled)	<80	98	<80	<80	<80	<80	<80
	Post-immune 3 & 4 (pooled)	<80	89	<80	<80	<80	<80	<80
	Post-immune 5	<80	125	<80	<80	<80	<80	<80
CH103 UCA het double knock-in / Repeated TF (TF Env in GLA-SE) (*n*=4)	Pre-bleed	<80	97	87	<80	<80	<80	<80
	Post-immune 1 & 2 (pooled)	<80	97	82	<80	<80	<80	89
	Post-immune 3 & 4 (pooled)	<80	<80	<80	<80	<80	<80	<80
	Post-immune 5	<80	93	<80	<80	<80	<80	<80
CH103 UCA het double knock-in / Sequential (CH505 Envs In GLA-SE) (*n*=5)	Pre-bleed	<80	95	<80	<80	<80	<80	<80
	Post-immune 1 & 2 (pooled)	<80	89	<80	<80	<80	<80	84
	Post-immune 3 & 4 (pooled)	<80	88	<80	<80	<80	<80	88
	Post-immune 5	<80	**208**	<80	<80	<80	<80	<80
C57BL/6 WT / - (naïve control)	n/a	<80	<80	<80	<80	<80	<80	<80

SUPPLEMENTARY METHODS

Recombinant mAb binding specificities. CH505 differentials were identified in the initial Ab screen (transient transfection) and/or verified upon testing as a mAb. CH505 differential binder mAb either 1) bound CH505 TF gp120 and not CH505 TF gp120 d371I, or 2) demonstrated ≥3-fold difference in binding to CH505 TF gp120 vs. Δ371I mutant when comparing the OD values during the linear phase of the binding curves for both Envs. CH505 differential binder mAbs with ≥3-fold difference in binding CH505 TF gp120 and d371I mutant generally blocked soluble (s) CD4 and CH106 binding to CH505 TF gp120 (5/5 mAbs tested). Eighteen antibodies that were identified as candidate CH505 differentials from the initial screen using small-scale IgG preparations were re-classified as CH505 non-differentials and non-HIV-1 Abs when screened as a purified mAb; 13/18 mAbs demonstrated ≤3 fold CH505 differential binding, and did not block sCD4 and CH106 binding to CH505 TF gp120, and 5/18 mAbs did not bind HIV-1 Env. Recombinant mAbs were assayed for polyreactivity based upon binding to a panel of >9400 human autoantigens as previously described (6). Non-autoreactive macaque mAb, Ab900564Rh, was used as a negative control. While Ab900564Rh demonstrated background binding levels ranging from 2 to 163 mean fluorescence intensity (MFI) with autoantigens bound by DH522 lineage antibodies, DH522 lineage antibodies bound autoantigens with high affinities ranging from ~3000-65000 MFI. Recombinant mAbs were tested for glycan binding in a custom glycan luminex microsphere assay previously described (2).

Expression of chimeric recombinant mAbs. Plasmids encoding the IGHV, IGKV, and IGLV genes were generated and used for recombinant mAb production in human embryonic kidney cell lines (ATCC , Manassas, VA) (7), via small-scale transfection and as purified mAbs in larger quantities (4). Purified rmAbs were dialyzed against PBS, analyzed, and stored at 4°C. Chimeric CH103, F105 and DH522.1 mAbs were generated by using the respective heavy or light chain gene plasmids from each of the three mAbs for 293i cell transfection using the standard protocol for recombinant mAb production referenced above.

Next generation sequencing of macaque Ig genes. Heavy and light chain immunoglobulin (Ig) repertoire next generation sequencing (NGS) of monkey RM-5556 was performed with the Illumina MiSeq platform utilizing primers targeting the V_H4 and $V_\lambda 2/3$ families to identify DH522 clonal members and profile the $V_\lambda 3$ repertoire using an NGS sequencing protocol previously described (8). To determine the germline gene segment candidate closest to human IGLV3-1, we performed sequential pairwise alignments between each of the 16,815 unique IGLV3 reads duplicated from two independent NGS runs and IGLV3-1 and chose the top-scoring sequences (191 apparently unrelated variable-region genes in total). From these, we inferred the most likely germline IGLV precursors using phylogenetic methods. The sequence identity of the closest RM5556 candidate IGLV germline gene to human IGLV3-1 was 85%.

Structural analysis of DH522 lineage Abs. We determined the crystal structures for each of the unliganded Fabs in the DH522 lineage: DH522UCA, DH522I1.2, DH522.1, and DH522.2 (see Supplementary Table 1, see Supplementary Figs. 4-5). To investigate how the mature antibodies in the DH522 lineage interact with the gp120 molecule, we also determined the crystal structure of DH522.2 Fab bound to a chimeric B.YU2 gp120 core (**Figure 3E**). The sequences of the antibodies exhibited low levels of mutation from germline (see Supplementary Fig. 5a). Accordingly, the Fab structures were largely invariant with a pair of mutations in HCDR3 standing out as the most potentially significant difference (see Supplementary Fig. 5b). In the DH522.2 complex structure, the HCDR3 His-Ser motif did not appear to be positioned to make significant contacts with gp120 itself. However, the corresponding Tyr-Asn motif in DH522.1, DH522UCA, and DH522IA could cause some unfavorable steric interactions with nearby residues in LCDR2 and gp120. The sterically smaller side chains of His-Ser better accommodate the allowed space within the paratope, resulting in a more optimized antibody-antigen interface and improved binding.

Statistical comparisons. Using the statistical analysis plan outlined in the methods, here we report the results of statistical comparisons for antibody immunogenetics [NHP79 – GLA-SE adjuvant; NHP88 – AS01E adjuvant]:

Mean IGHV nucleotide mutation frequencies (%):

CH505 differential binders

(1) CH505 TF Env - 3.3% (NHP79, N=3), undetected antibodies (NHP88, N=1); no statistical analysis done
(2) CH505 sequential Envs – 4.1% (NHP79, N=3), 5.1% (NHP88, N=3); *P=0.8*
(3) CH505 sequential or additive Envs – 4.1% (NHP79 seq., N=4), 3.9% (NHP79 add., N=4); P=1.0

CH505 non-differential binders

(1) CH505 TF Env – 6.4% (NHP79, N=3), 7.0% (NHP88, N=1); no statistical analysis done
(2) CH505 sequential Envs – 5.3% (NHP79, N=3), 7.3% (NHP88, N=3); *P=0.6*
(3) CH505 sequential or additive Envs – 5.3% (NHP79, N=4), 5.2% (NHP88, N=3); *P=1.0*

CH505 differential vs. non-differential binders vs. Non HIV-1-reactive (Total antibodies)

(1) CH505 differential binders, N=12 - 4.2%, CH505 non-differential binders, N=14 - 6.2%; *P=0.008*
(2) CH505 differential binders, N=12 - 4.2%, Non HIV-1-reactive, N=13 - 5.1%; *P=0.6*
(3) CH505 non-differential binders, N=14 - 6.2%, Non HIV-1-reactive, N=13 - 5.1%; *P=0.5*

Mean IGHV CDR3 length (amino acid length)

CH505 differential binders

(1) CH505 TF Env - 17 (NHP79, N=3), undetected antibodies (NHP88, N=1); no statistical analysis done
(2) CH505 sequential Envs – 16 (NHP79, N=3), 17 (NHP88, N=3); *P=1.*0
(3) CH505 sequential or additive Envs – 16 (NHP79 seq., N=4), 16 (NHP79 add., N=4); P=1.0

CH505 non-differential binders

(1) CH505 TF Env – 13 (NHP79, N=3), 14 (NHP88, N=1); no statistical analysis done
(2) CH505 sequential Envs – 15 (NHP79, N=3), 16 (NHP88, N=3); *P=0.6*
(3) CH505 sequential or additive Envs – 15 (NHP79, N=4), 14 (NHP88, N=3); *P=0.9*

CH505 differential vs. non-differential binders vs. Non HIV-1-reactive (Total antibodies)

(1) CH505 differential binders, N=12 - 17, CH505 non-differential binders, N=14 – 14; *P=0.008*
(2) CH505 differential binders, N=12 - 17, Non HIV-1-reactive, N=13 - 14; *P=0.008*
(3) CH505 non-differential binders, N=14 - 14, Non HIV-1-reactive, N=13 - 14; *P=0.5*

Frequency of CH505 differential binders (%) among all Abs (Env+ and non HIV-1-reactive antibodies) isolated per vaccine group

(1) CH505 TF Env - 13% (NHP79, N=3, macaque 5356 excluded for stats analysis), undetected antibodies (NHP88, N=1); no statistical analysis done
(2) CH505 sequential Envs – 8% (NHP79, N=3, macaque 5362 excluded from stats analysis), 13% (NHP88, N=3); *P=0.6*
(3) CH505 sequential or additive Envs – 8% (NHP79 seq., N=3, macaque 5362 excluded from stats analysis), 14% (NHP79 add., N=4); *P=0.6*
(4) [NHP79, N=6 (macaque 5356 and macaque 5362 excluded from stats analysis) + NHP88, N=4] CH505 TF vs. sequential Envs – 9.5% (TF alone), 11% (Seq.); *P=1.0*
(5) [NHP79, N=10 (macaque 5356 and macaque 5362 excluded from stats analysis) + NHP88, N=4] CH505 TF vs. sequential and additive Envs – 9.5% (TF alone), 12% (Seq.+Add.); *P=0.9*

Generation of CH103UCA knock-in mouse models. Mice with homozygously (hom) knocked-in CH103UCA V_HDJ_H rearrangements ($VDJ^{+/+}$ knock-in strains), or heterozygously (het) or homozygously knocked-in CH103UCA V_HDJ_H rearrangements and heterozygous $V\lambda J\lambda$ rearrangements, i.e. $VDJ^{+/-}$x $VJ^{+/-}$ (het double knock-in) and $VDJ^{+/+}$ x $VJ^{+/-}$ (double knock-in) strains, respectively, were generated on the C57BL/B6 background, based on Ig site-directed gene-targeting techniques previously described for engineering knock-in models expressing the original (mature) 2F5 and 4E10 bnAb rearrangements (9-11). Briefly, CH103UCA $V_HDJ_H^{+/-}$ knock-in mice were first generated (see Supplementary Fig. 7) by knocking in the published V_HDJ_H rearrangement of the inferred CH103UCA (4), via replacement of the mouse J_H cluster with the CH103UCA V_HDJ_H expression cassette (comprised of J558 H10 promoter+split leader and CH103UCA V_HDJ_H rearrangement sequences), using previously described murine HC locus targeting constructs and strategies (9).

In parallel, recombinant ES cells bearing the murine kappa locus-targeted inferred CH103UCA $V_\lambda J_\lambda$ rearrangement sequence (4) were generated by replacing $J_\kappa 1$ and $J_\kappa 2$ with the CH103UCA $V_\lambda J_\lambda$ cassette (comprised of the VκOx1 promoter+split leader and pre-rearranged CH103 UCA VλJλ segments), also based on previously published methods (10), to derive CH103UCA $V\lambda J\lambda^{+/-}$ knock-in mice (see Supplementary Fig. 7). Although the CH103UCA VλJλ is normally expressed at the human Igλ locus, we targeted it into the mouse Igκ, with two considerations in mind: i) we sought to retain the ability of CH103UCA knock-in models to undergo extensive secondary LC rearrangement events more resembling those at the more diverse human λ locus, relative to the much more restricted mouse λ locus; thus by targeting the CH103UCA VλJλ rearrangement specifically at mouse Igκ1/2, our targeting strategy ensures secondary rearrangement events involving many possible upstream V_L families to downstream Jκ4/Jκ5 elements can occur; hence preserving the potential for receptor editing *in cis* at the knock-in allele to remove any potential tolerizing self-reactivity inherent to the UCA, and ii) Ig expression at mouse Igλ is poor, relative to that at human Igλ and mouse/human Igκ (12). Prior to generating the CH103 UCA $V_\lambda J_\lambda^{+/-}$ knock-in mice, specificity of the CH103UCA Ab when bearing a CH1013UCA $V_\lambda J_\lambda$ rearrangement linked to a Cλ (a Cκ region) was tested by generating both versions as recombinant Abs and demonstrating equivalent ability to bind autologous TF Env or heterologous Env in ELISAs (see Supplementary

Fig. 7). Finally, CH103UCA double knock-in and het double knock-in mice were generated by cross-breeding CH103UCA $V_HD_HJ_H^{+/+}$ and CH103 UCA $V_\lambda J_\lambda^{+/+}$ mice, also as described (10).

Neutralization activity measurement of CH103 het dKI serum. Mouse plasma Abs were screened for neutralization by adapting the well-established TZM-bl HIV-1 pseudo-virus infectivity neutralization assay (13) for measurement of mouse serum, using appropriate negative controls, including non-KI (WT BL/6) sera, the non-HIV pseudovirus SVA-MuLV, all as previously detailed (14).

Sequence analysis of mouse antibody genes. Using the RT-PCR isolation and sequence analysis methods described above, here we report the immunogenetics of VDJ/VJ pairs recovered from single differential-sorted (mutant Δ371 TF Env-, WT TF Env+) IgG^+ memory B-cells (gated as live, $B220^+CD19^+CD93^-CD38^+IgG^+$,$IgM^-$,$IgD^-$) splenocytes, isolated either 10d after 2nd repeated (TF Env x2) immunizations, sequential (TF+week 53 Env) immunizations, or control (saline or adjuvant-only) immunizations performed in CH103 het double knock-in ($V_HDJ_H^{+/-}$ x $V\lambda J\lambda^{+/-}$) mice:

Saline-injected control mice (44 pairs total)

1) Pairs expressing both knock-in HC and knock-in LC rearrangements (*1*)
2) Pairs expressing the knock-in HC rearrangement with a murine endogenous VκJκ rearrangement (*23*), the latter distributed as follows: Vκ5-39 Jκ5 (*14*), Vκ5-39 Jκ2 (*3*), Vκ5-39 Jκ4 (*2*), Vκ5-39 Jκ1 (*1*), Vκ5-43 Jκ2 (*1*), Vκ4-69 Jκ5 (*1*), and Vκ4-78 Jκ5 (*1*)
3) Pairs expressing a murine endogenous V_HDJ_H rearrangement with the knock-in LC rearrangement (*5*), the former all unique rearrangements: V_H1-50 D_H4-1 J_H4, V_H1-55 D_H1-1 J_H2, V_H1-26 D_H2-2 J_H2, V_H1-80 D_H2-3 J_H2, and V_H1-85 D_H3-3 J_H3
4) Pairs expressing murine endogenous V_HDJ_H and VκJκ rearrangements (*15*), all expressing unique HC and LC rearrangements, except for two pairs, both expressing V_H1-9 D_H2-2 J_H2 / Vκ6-17 Jκ4

GLA-SE-injected control mice (41 pairs total)

1) Pairs expressing both knock-in HC and knock-in LC rearrangements (*0*)
2) Pairs expressing the knock-in HC rearrangement with a murine endogenous VκJκ rearrangement (*3*), the latter distributed as follows: Vκ5-39 Jκ1 (*2*) and Vκ5-39 Jκ5 (*1*)
3) Pairs expressing a murine endogenous V_HDJ_H rearrangement with the knock-in LC rearrangement (*0*)
4) Pairs expressing murine endogenous V_HDJ_H and VκJκ rearrangements (*38*), all expressing unique HC and LC rearrangements, except for four pairs, two both expressing V_H8-12 D_H2-3 J_H3 / Vκ19-93 Jκ1, and two both expressing V_H1-82 D_H1-1 J_H2 / Vκ6-25 Jκ2

Repeated (TF Env x2) immunized mice (52 pairs total)

1) Pairs expressing both knock-in HC and knock-in LC rearrangements (*4*)

2) Pairs expressing the knock-in HC rearrangement with a murine endogenous VκJκ rearrangement (*10*), the latter distributed as follows: Vκ5-39 Jκ5 (*7*), Vκ5-39 Jκ4 (*1*), Vκ5-39 Jκ1 (*1*), and Vκ8-30 Jκ4 (*1*)

3) Pairs expressing a murine endogenous $V_H DJ_H$ rearrangement with the knock-in LC rearrangement (*16*), the former distributed as follows: V_H1-22 D_H4-1 J_H3 (*2*), V_H1-39 D_H1-1 J_H2 (*1*), V_H1-75 D_H4-1 J_H2 (*1*), V_H1-26 D_H2-2 J_H3 (*1*), V_H1-53 D_H1-1 J_H2 (*1*), V_H1-76 D_H1-1 J_H3 (*1*), V_H1-26 D_H1-1 J_H2 (*1*), V_H6-6 D_H2-2 J_H4 (*1*), V_H1-55 D_H3-1 J_H2 (*1*), V_H4-1 D_H1-1 J_H2 (*1*), V_H2-3 D_H1-1 J_H1 (*1*), V_H1-19 D_H2-4 J_H3 (*1*), V_H1-5 D_H3-3 J_H4 (*1*), V_H3-6 D_H2-4 J_H4 (*1*), and V_H12-3 D_H2-3 J_H3 (*1*)

4) Pairs expressing murine endogenous $V_H DJ_H$ and VκJκ rearrangements (*22*), all expressing unique HC and LC rearrangements

Sequential (TF+week 53 Env) immunized mice (44 pairs total)

1) Pairs expressing both knock-in HC and knock-in LC rearrangements (*4*)

2) Pairs expressing the knock-in HC rearrangement with a murine endogenous VκJκ rearrangement (*8*), the latter distributed as follows: Vκ5-39 Jκ5 (*3*), Vκ5-39 Jκ1 (*2*), Vκ14-111 Jκ5 (*1*), Vκ3-12 Jκ5 (*1*), and Vκ4-80 Jκ1 (*1*)

3) Pairs expressing a murine endogenous $V_H DJ_H$ rearrangement with the knock-in LC rearrangement (*13*), the former all unique rearrangements: V_H14-12 D_H2-12 J_H2, V_H1-64 D_H2-2 J_H4, V_H1-34 D_H2-4 J_H2, V_H14-2 D_H6-2 J_H2, V_H1-53 D_H2-3 J_H2, V_H8-8 D_H2-4 J_H4, V_H1-9 D_H2-4 J_H3, V_H6-6 D_H2-3 J_H2, V_H3-6 D_H1-2 J_H3, V_H1-34 D_H2-5 J_H2, V_H1-53 D_H1-1 J_H1, V_H2-9 D_H1-1 J_H2, and V_H1-18 D_H2-13 J_H2

4) Pairs expressing murine endogenous $V_H DJ_H$ and VκJκ rearrangements (*19*), all expressing unique HC and LC rearrangements

SUPPLEMENTARY REFERENCES

1. Zhou T, Doria-Rose NA, Cheng C, Stewart-Jones GBE, Chuang GY, Chambers M, Druz A, Geng H, McKee K, Kwon YD, O'Dell S, Sastry M, Schmidt SD, Xu K, Chen L, Chen RE, Louder MK, Pancera M, Wanninger TG, Zhang B, Zheng A, Farney SK, Foulds KE, Georgiev IS, Joyce MG, Lemmin T, Narpala S, Rawi R, Soto C, Todd JP, Shen CH, Tsybovsky Y, Yang Y, Zhao P, Haynes BF, Stamatatos L, Tiemeyer M, Wells L, Scorpio DG, Shapiro L, McDermott AB, Mascola JR, Kwong PD. Quantification of the Impact of the HIV-1-Glycan Shield on Antibody Elicitation. Cell Rep. 2017;19(4):719-32. doi: 10.1016/j.celrep.2017.04.013. PubMed PMID: 28445724.

2. Saunders KO, Nicely NI, Wiehe K, Bonsignori M, Meyerhoff RR, Parks R, Walkowicz WE, Aussedat B, Wu NR, Cai F, Vohra Y, Park PK, Eaton A, Go EP, Sutherland LL, Scearce RM, Barouch DH, Zhang R, Von Holle T, Overman RG, Anasti K, Sanders RW, Moody MA, Kepler TB, Korber B, Desaire H, Santra S, Letvin NL, Nabel GJ, Montefiori DC, Tomaras GD, Liao HX, Alam SM, Danishefsky SJ, Haynes BF. Vaccine Elicitation of High Mannose-Dependent Neutralizing Antibodies against the V3-Glycan Broadly Neutralizing Epitope in Nonhuman Primates. Cell Rep. 2017;18(9):2175-88. doi: 10.1016/j.celrep.2017.02.003. PubMed PMID: 28249163.

3. McNicholas S, Potterton E, Wilson KS, Noble ME. Presenting your structures: the CCP4mg molecular-graphics software. Acta Crystallogr D Biol Crystallogr. 2011;67(Pt 4):386-94. Epub 2011/03/18. doi: 10.1107/S0907444911007281. PubMed PMID: 21460457; PMCID: PMC3069754.

4. Liao HX, Lynch R, Zhou T, Gao F, Alam SM, Boyd SD, Fire AZ, Roskin KM, Schramm CA, Zhang Z, Zhu J, Shapiro L, Mullikin JC, Gnanakaran S, Hraber P, Wiehe K, Kelsoe G, Yang G, Xia SM, Montefiori DC, Parks R, Lloyd KE, Scearce RM, Soderberg KA, Cohen M, Kamanga G, Louder MK, Tran LM, Chen Y, Cai F, Chen S, Moquin S, Du X, Joyce MG, Srivatsan S, Zhang B, Zheng A, Shaw GM, Hahn BH, Kepler TB, Korber BT, Kwong PD, Mascola JR, Haynes BF, Program NCS. Co-evolution of a broadly neutralizing HIV-1 antibody and founder virus. Nature. 2013;496(7446):469-76. doi: 10.1038/nature12053. PubMed PMID: 23552890; PMCID: PMC3637846.

5. Rogozin IB, Diaz M. Cutting edge: DGYW/WRCH is a better predictor of mutability at G:C bases in Ig hypermutation than the widely accepted RGYW/WRCY motif and probably reflects a two-step activation-induced cytidine deaminase-triggered process. J Immunol. 2004;172(6):3382-4. PubMed PMID: 15004135.

6. Yang G, Holl TM, Liu Y, Li Y, Lu X, Nicely NI, Kepler TB, Alam SM, Liao HX, Cain DW, Spicer L, VandeBerg JL, Haynes BF, Kelsoe G. Identification of autoantigens recognized by the 2F5 and 4E10 broadly neutralizing HIV-1 antibodies. J Exp Med. 2013;210(2):241-56. doi: 10.1084/jem.20121977. PubMed PMID: 23359068; PMCID: PMC3570098.

7. Liao HX, Levesque MC, Nagel A, Dixon A, Zhang R, Walter E, Parks R, Whitesides J, Marshall DJ, Hwang KK, Yang Y, Chen X, Gao F, Munshaw S, Kepler TB, Denny T, Moody MA, Haynes BF. High-throughput isolation of immunoglobulin genes from single human B cells and expression as monoclonal antibodies. J Virol Methods. 2009;158(1-2):171-9. doi: 10.1016/j.jviromet.2009.02.014. PubMed PMID: 19428587; PMCID: PMC2805188.

8. Zhang R, Verkoczy L, Wiehe K, Munir Alam S, Nicely NI, Santra S, Bradley T, Pemble CW, Zhang J, Gao F, Montefiori DC, Bouton-Verville H, Kelsoe G, Larimore K, Greenberg PD, Parks R, Foulger A, Peel JN, Luo K, Lu X, Trama AM, Vandergrift N, Tomaras GD, Kepler TB, Moody MA, Liao HX, Haynes BF. Initiation of immune tolerance-controlled HIV gp41 neutralizing B cell lineages. Sci Transl Med. 2016;8(336):336ra62. doi: 10.1126/scitranslmed.aaf0618. PubMed PMID: 27122615; PMCID: PMC5006673.

9. Verkoczy L, Diaz M, Holl TM, Ouyang YB, Bouton-Verville H, Alam SM, Liao HX, Kelsoe G, Haynes BF. Autoreactivity in an HIV-1 broadly reactive neutralizing antibody variable region heavy chain induces

immunologic tolerance. Proc Natl Acad Sci U S A. 2010;107(1):181-6. doi: 10.1073/pnas.0912914107. PubMed PMID: 20018688; PMCID: PMC2806760.

10. Verkoczy L, Chen Y, Bouton-Verville H, Zhang J, Diaz M, Hutchinson J, Ouyang YB, Alam SM, Holl TM, Hwang KK, Kelsoe G, Haynes BF. Rescue of HIV-1 broad neutralizing antibody-expressing B cells in 2F5 VH x VL knockin mice reveals multiple tolerance controls. J Immunol. 2011;187(7):3785-97. doi: 10.4049/jimmunol.1101633. PubMed PMID: 21908739; PMCID: PMC3192533.

11. Chen Y, Zhang J, Hwang KK, Bouton-Verville H, Xia SM, Newman A, Ouyang YB, Haynes BF, Verkoczy L. Common tolerance mechanisms, but distinct cross-reactivities associated with gp41 and lipids, limit production of HIV-1 broad neutralizing antibodies 2F5 and 4E10. J Immunol. 2013;191(3):1260-75. doi: 10.4049/jimmunol.1300770. PubMed PMID: 23825311; PMCID: PMC3725147.

12. Alt FW, Oltz EM, Young F, Gorman J, Taccioli G, Chen J. VDJ recombination. Immunol Today. 1992;13(8):306-14. doi: 10.1016/0167-5699(92)90043-7. PubMed PMID: 1510813.

13. Montefiori DC, Karnasuta C, Huang Y, Ahmed H, Gilbert P, de Souza MS, McLinden R, Tovanabutra S, Laurence-Chenine A, Sanders-Buell E, Moody MA, Bonsignori M, Ochsenbauer C, Kappes J, Tang H, Greene K, Gao H, LaBranche CC, Andrews C, Polonis VR, Rerks-Ngarm S, Pitisuttithum P, Nitayaphan S, Kaewkungwal J, Self SG, Berman PW, Francis D, Sinangil F, Lee C, Tartaglia J, Robb ML, Haynes BF, Michael NL, Kim JH. Magnitude and breadth of the neutralizing antibody response in the RV144 and Vax003 HIV-1 vaccine efficacy trials. J Infect Dis. 2012;206(3):431-41. doi: 10.1093/infdis/jis367. PubMed PMID: 22634875; PMCID: PMC3392187.

14. Verkoczy L, Chen Y, Zhang J, Bouton-Verville H, Newman A, Lockwood B, Scearce RM, Montefiori DC, Dennison SM, Xia SM, Hwang KK, Liao HX, Alam SM, Haynes BF. Induction of HIV-1 broad neutralizing antibodies in 2F5 knock-in mice: selection against membrane proximal external region-associated autoreactivity limits T-dependent responses. J Immunol. 2013;191(5):2538-50. doi: 10.4049/jimmunol.1300971. PubMed PMID: 23918977; PMCID: PMC3870053.
